# A cross-strain CRISPRi sgRNA library for *Streptococcus agalactiae*

**DOI:** 10.1128/mra.00521-26

**Published:** 2026-06-04

**Authors:** William D. Cutts, Austin D. Sandobal, Aidan W. Flanagan, Brice K. Gorman, Brandon J. Kim

**Affiliations:** 1 Department of Biological Sciences, University of Texas at Dallas, Dallas, Texas, USA; 2 Department of Biological Sciences, University of Alabama, Tuscaloosa, Alabama, USA; 3 Department of Microbiology, Heersink School of Medicine, University of Alabama at Birmingham, Birmingham, Alabama, USA; Indiana University Bloomington, Bloomington, Indiana, USA

**Keywords:** group B streptococcus, CRISPR interference, sgRNA, library

## Abstract

We present a comprehensive, *in silico*-designed CRISPR interference (CRISPRi) sgRNA database targeting the hypervirulent *Streptococcus agalactiae* strain COH1. This resource provides broad, redundant, and cross-strain functional coverage, enabling scalable CRISPRi-based interrogation of conserved and strain-specific determinants of GBS biology, colonization, and pathogenesis.

## ANNOUNCEMENT

*Streptococcus agalactiae* (Group B Streptococcus; GBS) is a commensal of the gastrointestinal and vaginal microbiota and an opportunistic pathogen capable of causing invasive disease, including sepsis, pneumonia, and meningitis (1–4). GBS remains the leading cause of neonatal bacterial meningitis worldwide following transmission to immunologically naïve neonates (3). Functional characterization of GBS colonization, persistence, and virulence determinants has traditionally relied on gene disruption approaches that are often labor-intensive and challenging to scale (3, 5–13).

CRISPR interference (CRISPRi) provides a rapid, precise alternative for functional genetic interrogation by employing a catalytically inactive Cas9 (dCas9) to repress transcription of targeted genes (14, 15). CRISPRi knockdown strength is tunable and inversely correlated with the distance between the single-guide RNA (sgRNA) binding site and the transcriptional start site (TSS) (15, 16). We, therefore, designed an *in silico* CRISPRi sgRNA library to facilitate scalable GBS gene function investigation (17).

**Fig 1:**
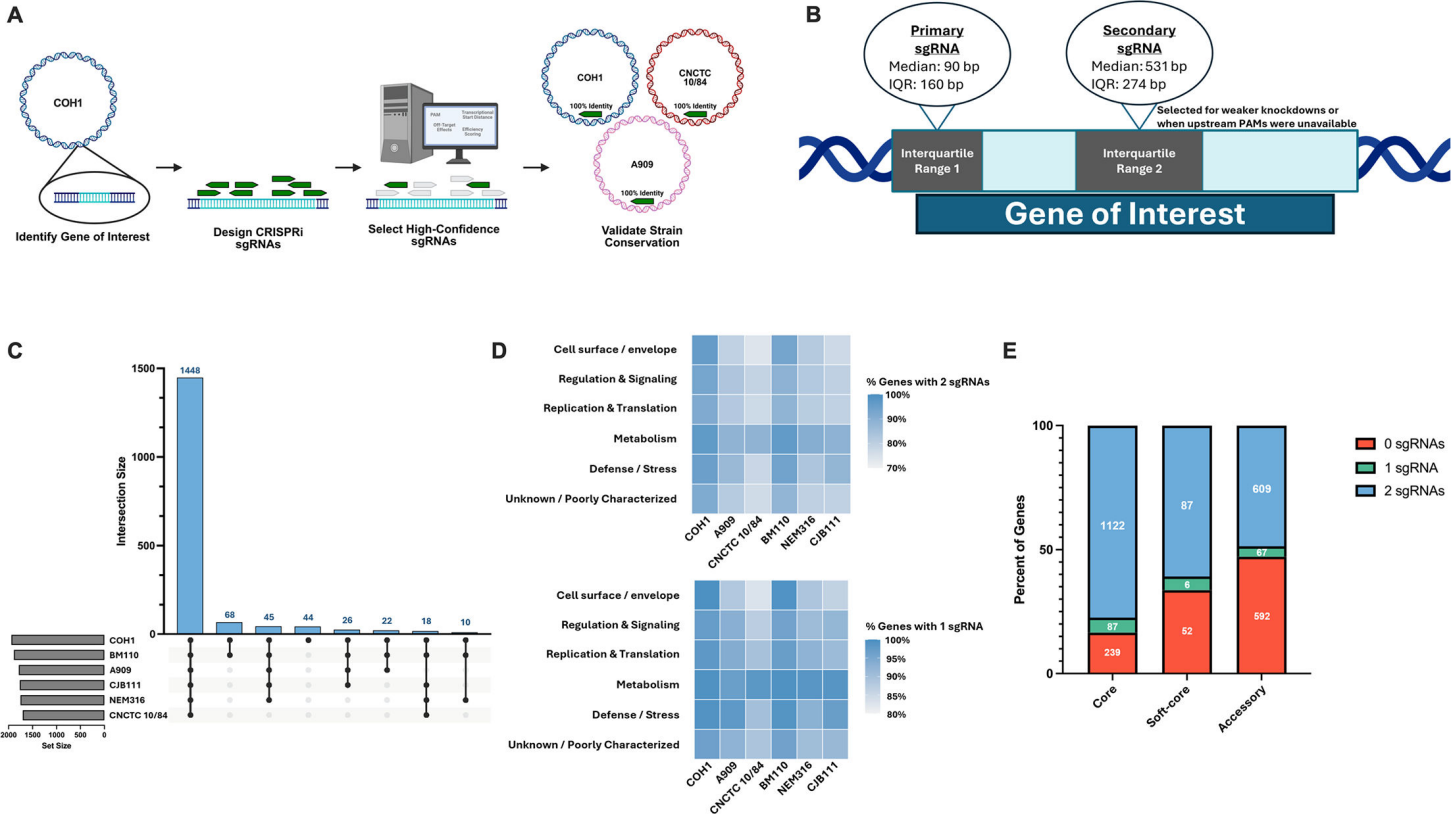
Design and functional coverage of a CRISPRi sgRNA library for *Streptococcus agalactiae*. (**A**) Overview of the *in silico* sgRNA design workflow. sgRNAs were designed against the COH1 reference genome using CHOPCHOP to maximize predicted on-target efficiency and minimize off-target effects. (**B**) Distribution of sgRNA target positions relative to gene start codons. For each gene, a primary sgRNA was selected proximal to the transcriptional start site to achieve stronger knockdown, and a secondary sgRNA was positioned further downstream to enable weaker repression where upstream PAMs were unavailable. (**C**) Intersection of genes targeted by at least one conserved sgRNA across six commonly used GBS strains (COH1, A909, CNCTC 10/84, NEM316, BM110, and CJB111). (**D**) Heatmaps depicting functional coverage of the sgRNA library across major biological categories shown as the percentage of genes with two sgRNAs (robust coverage) or at least one sgRNA (overall accessibility) in each strain. (**E**) Core genome coverage determined using Roary. The proportion of genes conserved across all six strains that are targeted by one or more sgRNAs is shown, demonstrating broad coverage of the GBS core genome. Core is defined as shared among all strains; soft-core is conserved in five of the strains, and accessory is conserved in four or fewer strains.

We generated a database of 3,595 sgRNAs targeting 1,944/2,073 annotated protein-coding genes in the hypervirulent GBS strain COH1, excluding rRNA and tRNA loci. sgRNAs were designed using CHOPCHOP v3.0, a tool previously utilized in streptococcal and enterococcal CRISPRi sgRNA design, to maximize predicted on-target efficiency and minimize off-target effects (Fig. 1A) (18–20). While CHOPCHOP scoring frameworks are primarily derived from eukaryotic data sets, the design criteria used emphasize sequence specificity and PAM compatibility, which are broadly applicable to bacterial CRISPRi systems. sgRNA activity may vary in GBS and should be empirically validated for individual targets (21, 22).

Where possible, two sgRNAs were designed per gene to provide redundancy and exploit CRISPRi tunability: a primary sgRNA positioned proximally to the TSS to achieve stronger repression (median 90 bp, interquartile range [IQR] 160 bp) and a secondary sgRNA positioned downstream to produce a weaker knockdown (median 541 bp, IQR 274 bp) (Fig. 1B). While this does not provide the saturation achieved by larger CRISPRi libraries, the inclusion of two guides per gene enables initial investigation while supporting essential gene interrogation, for which partial knockdown may be required (17). Although alternative approaches, including mismatch-based sgRNA tuning, have been described, the positional strategy employed here provides a simple, scalable framework for modulating repression strength across the genome.

Although sgRNA design was prioritized for COH1, we sought to maximize the utility of this resource across commonly used GBS laboratory strains: A909, CNCTC 10/84, NEM316, BM110, and CJB111 (17). A total of 1,448/2,073 target genes possess at least one sgRNA conserved across these strains (Fig. 1C). Functional coverage of the library was assessed using EggNOG Mapper v2.1.13 with default parameters to assign genes to COG functional categories, revealing redundancy across biological processes (Fig. 1D) (23, 24). Core genome coverage was evaluated using Roary v3.13 with a 90% amino acid identity threshold and a strict core definition requiring gene presence in 100% of strains. Of 1,448 core genes (excluding rRNAs and tRNAs), 1,319 were targeted by at least one sgRNA (Fig. 1E) (25).

Together, this CRISPRi sgRNA database provides a versatile, accessible resource for targeted functional genomics in GBS, enabling rapid prioritization and interrogation of conserved and strain-specific colonization and pathogenesis determinants.

## Data Availability

The CRISPRi sgRNA library generated in this study has been deposited in Figshare and is publicly available under DOI 10.6084/m9.figshare.31362961.
